# Barriers and Proposed Solutions to a Successful Implementation of Pediatric Sepsis Protocols

**DOI:** 10.3389/fped.2021.755484

**Published:** 2021-11-10

**Authors:** Daniela Nasu Monteiro Medeiros, Audrey Ogawa Shibata, Cristiane Freitas Pizarro, Maria de Lourdes Alves Rosa, Marta Pessoa Cardoso, Eduardo Juan Troster

**Affiliations:** ^1^Pediatric Intensive Care Unit, Hospital Israelita Albert Einstein, São Paulo, Brazil; ^2^Health Practices Department, Hospital Municipal Dr Moysés Deutsch - M‘Boi Mirim, São Paulo, Brazil; ^3^Faculdade Israelita de Ciências em Saúde, Hospital Albert Einstein, São Paulo, Brazil

**Keywords:** protocol and guidelines, children, mortality, implementation, outcomes, barriers

## Abstract

The implementation of managed protocols contributes to a systematized approach to the patient and continuous evaluation of results, focusing on improving clinical practice, early diagnosis, treatment, and outcomes. Advantages to the adoption of a pediatric sepsis recognition and treatment protocol include: a reduction in time to start fluid and antibiotic administration, decreased kidney dysfunction and organ dysfunction, reduction in length of stay, and even a decrease on mortality. Barriers are: absence of a written protocol, parental knowledge, early diagnosis by healthcare professionals, venous access, availability of antimicrobials and vasoactive drugs, conditions of work, engagement of healthcare professionals. There are challenges in low-middle-income countries (LMIC). The causes of sepsis and resources differ from high-income countries. Viral agent such as dengue, malaria are common in LMIC and initial approach differ from bacterial infections. Some authors found increased or no impact in mortality or increased length of stay associated with the implementation of the SCC sepsis bundle which reinforces the importance of adapting it to most frequent diseases, disposable resources, and characteristics of healthcare professionals. Conclusions: (1) be simple; (2) be precise; (3) education; (5) improve communication; (5) work as a team; (6) share and celebrate results.

## Introduction

Sepsis is a major childhood disease in terms of frequency and severity (1). The incidence of sepsis is increasing in the pediatric population, mainly due to the higher survival of very low-birth-weight infants and children with chronic conditions (1). Studies have shown that 30–62% of patients are currently treated according to guidelines. The implementation of managed protocols contributes to a systematized approach and to a continuous evaluation of results, always focusing on improving clinical practice and outcomes (2–4). Protocols might help clinicians treat sepsis because they make a systematic approach, early diagnosis, and treatment easier and through. But how to implement a pediatric sepsis protocol successfully?

This is a narrative review that includes the history of sepsis protocols, the importance of its utilization, barriers to implementation, and proposed solutions.

## Definitions and History of Protocols in Pediatric Sepsis

Guidelines are communications, developed by methodology, to support the best care of a specific disease (5).

Bundle is a concept developed by the Institute of Healthcare Improvement (IHI). It is a small set of evidence-based interventions for a defined patient segment/population and care setting that, when implemented together, will result in significantly better outcomes than when applied individually (6).

In 2001, IHI developed the concept of a bundle at a partnership with 13 hospitals to rethink intensive care and to discover how to achieve the highest levels of reliability in critical care processes. The bundle design guidelines are as follows: (1) The bundle has three to five interventions (elements), with strong clinician agreement; (2) Each bundle element is relatively independent; (3) The bundle is used with a defined patient population in one location; (4) The multidisciplinary care team develops the bundle; (5) Bundle elements should be descriptive rather than prescriptive, to allow for local customization and appropriate clinical judgment. According to IHI, success is not only related to following the steps of a care bundle but to the redesign of work processes, to improve communication strategies, and infrastructure, along with sustained measurement and vigilance (6).

In 2003, the Surviving Sepsis Campaign (SSC) started a partnership with IHI to disseminate sepsis bundles for resuscitation and management in adult patients (6, 7).

In 2014, The SSC for children recommended that institutions participate in the process by adopting recognition, resuscitation, management, and performance bundles, as follows: (1) recognition bundle to screen patients with sepsis, collect laboratory exams and blood culture, and initiate treatment within 15 min of diagnosis; (2) resuscitation bundle to start crystalloid infusion within 30 min and antibiotics and vasoactive drugs within 60 min of diagnosis; (3) Management bundle with multimodal monitoring; (4) a performance bundle to provide guidance on measuring adherence to recognition, resuscitation, and stabilization bundles. In addition, there is a recommendation to apply root cause analysis to identify adherence barriers (8).

Finally, the 2020 SSC panel supports that these guidelines should constitute a general scheme of “best practice,” but that translation to treatment algorithms or bundles and standards of care will need to account for variation in the availability of local healthcare resources (9).

## Improving Performance Through Quality Improvement Tools

The first step to implement a protocol is the elaboration of a written protocol. Surveys applied in France and Saudi Arabia to pediatric intensivists showed that an absence of a written protocol was one of greatest barrier to adoption of systematic treatment according to SSC guidelines (10, 11).

For protocol elaboration and measurement of performance, there are quality-improvement (QI) tools such as Ishikawa diagram and plan-do-study-act (PDSA) or plan-do-check-act (PDCA) cycles.

Ishikawa diagram is a good diagnostic tool to apply root-cause analysis. Cruz et al. applied a root-cause analysis to identify barriers to SSC guidelines and began a QI project named “Shock Protocol” that helped to re-think and re-design the process of treatment (12).

Rodrigues-Santos et al. used PDCA improvement cycles to implement protocol in a pediatric hospital in Brazil, which resulted in some of the following actions: (a) availability of crystalloid stock in the inpatient unit; (b) implementation of an assistance sheet to guide the steps of the 1-h bundle; (c) standardization of antibiotic regimens for the treatment of sepsis; (d) implementation of a manual antibiotic prescription sheet to enable the immediate dispensation of the drug; (e) provision of a device for remote activation of the attending physician in cases of suspected sepsis; (f) distribution of posters presenting the protocol, clarifying the diagnostic criteria, and the objectives to be achieved during the first hour; (g) distribution of cards for healthcare professionals, with ranges of normal vital signs by age group and warning signs for sepsis recognition; (h) training the multidisciplinary team in simulations with patients with suspected sepsis every 6 months; (i) scheduling of a semiannual meeting to disseminate sepsis care indicators (13).

Long et al. also implemented a local sepsis guideline at a Melbourne, Australia. The group identified quality gaps in ED sepsis management through sentinel events reviewed during monthly departmental morbidity and mortality meetings, and a local guideline was developed through a Clinical Practice Guideline Committee, comprising representatives from state-wide critical care and medical teams (14).

Plan-do-study-act is a tool to implement improvement cycles. Damiani et al. performed a systematic review of 50 studies in an adult population evaluating the impact of performance improvement programs on compliance with the SSC guideline-based bundles and/or mortality. They concluded that, despite high inconsistency across studies, performance improvement programs were associated with increased compliance with the complete 6-h bundle (*OR* = 4.12 [95% confidence interval 2.95–5.76], *I*^2^ = 87.72%, *k* = 25, *N* = 50,081) and the complete 24-h bundle (*OR* = 2.57 [1.74–3.77], *I*^2^ = 85.22%, *k* = 11, *N* = 45,846) and with a reduction in mortality (*OR* = 0.66 [0.61–0.72], *I*^2^ = 87.93%, *k* = 48, *N* = 434,447) (15).

A multidisciplinary team is important to any QI initiative. Cruz et al. pointed that leadership support and the work of a multidisciplinary team with emergence department (ED) and pediatric intensive care unit (PICU) physicians and nurses were important for implementation. The team identified obstacles such as variability in experience of staff in performing initial evaluations; lack of adequate nursing staff for resource-intensive patients; difficulty obtaining frequent vital-sign measurements; lack of standardization of empiric antibiotics and diagnostic tests; lack of medication prioritization; and barriers to patient flow through the institution (12).

Lamba et al. implemented multiples PDSA cycles to increase compliance to late onset sepsis and antimicrobial stewardship in a neonatal intensive care unit (NICU) in Tampa (USA). A multidisciplinary team was formed and included physicians (neonatology fellows and attendings, infectious disease attendings), nurse practitioners, nurses, nurse educators, pharmacists, administrators (NICU nurse manager, NICU medical director), business intelligence, and information technology. They achieved ≥75% compliance, after interventions. The authors identified Key elements for successful implementation: (1) team composition, (2) meeting huddle efficiency, and (3) providing actionable feedback and education on identified opportunities following best practices for QI (16).

## Results of Protocolized Treatment

Paul et al., Fernandez-Sarmiento et al., Long et al., and Cruz et al. found a reduction in starting time of fluids and antibiotics administration after protocol implementation and education (2, 12, 14, 17).

Ackan Arikan et al. reported a reduction on kidney dysfunction, and Balamuth et al. related reduction on organ dysfunction after starting a protocolized treatment (18, 19).

Larsen et al. observed a reduction on length of stay (20).

In studies of Evans et al. on New York State and Rodrigues-Santos et al. in Brazil, there was reduction on mortality (13, 21).

## Barriers and Solutions

### Parental Knowledge

Ideally, parents should be educated to be able to recognize sepsis signs. Launay et al. analyzed the frequency and types of suboptimal care and medical errors in 21 children who died of severe bacterial infection in France. Parental delay in seeking medical care was identified in 33% of cases (22). Kang et al. surveyed 101 hospitals in 41 countries associated with the World Federation of Adult and Pediatric Intensive Care Societies. Parental education for the early recognition of sepsis was pointed as one of the biggest barriers to treatment (23). Moretti et al. interviewed 1986 people walking in a park in Porto Alegre (Brazil) to assess knowledge of sepsis and acute myocardial infarction. The researchers found that only 19.1% of respondents had some knowledge about sepsis, while 97.4% of respondents had knowledge about acute myocardial infarction (24).

Larsen et al. conducted a multicenter quality improvement (QI) learning collaborative of 56 US children's hospitals to reduce mortality and hospital-onset sepsis. The group developed a key driver diagram (KDD) based on treatment guidelines, available evidence, and expert opinion. One of the KDD primary drivers was engagement of patients and families. This bundle was composed by the following items: (1) include family in planning and implementing teams; (2) family-activated rapid response system; (3) real-time discussions with family members to explain what happened, provide education, answer questions, and identify concerns about the patient's care; (4) create or engage organization, service line, and/or care setting patient and family engagement resources; (5) ensure common understanding of patient and family engagement across the organization (25).

### Diagnoses by Healthcare Professionals

The diagnosis of sepsis and septic shock is essentially clinical, and survival depends on three main factors: (1) early recognition/identification of patients with suspected sepsis and septic shock; (2) immediate institution of aggressive and staggered treatment; (3) attentive clinical, laboratory, and hemodynamic monitoring. Shock should be recognized before hypotension occurs using clinical criteria.

However, it is a difficult diagnosis for healthcare professionals. Per example, in children, the normal range of vital signals varies according to the age group. Furthermore, there was no recent review of diagnostic criteria for sepsis/septic shock. The possibility of septic shock in pediatric patients should be considered when there is infection associated with clinical signs of inadequate tissue perfusion. Different interpretations are common between healthcare professionals (26, 27). Launay et al. reported that delay on sepsis recognition by healthcare professionals is one of the causes of suboptimal care (22).

The recognition package aims to achieve a rapid identification of the patient with sepsis and septic shock and should have a tool that allows the activation of the medical evaluation and the resuscitation package within 15 min (8). A solution for this quick trigger could be an alert system on triage or on electronic medical chart and artificial intelligence application (28, 29).

Systematic screening needs to be adapted according to the patient's profile and available resources and procedures within each institution. Evaluation for the effectiveness and sustainability of screening should be incorporated as part of this process. In other words, protocols need to be individualized, and for their success, it is necessary to implement education, training, and the involvement of a multidisciplinary care team composed of physicians, nurses, clinical pharmacists amongst others.

Therefore, each institution must develop and adopt its own protocol, and this must be guided by goals, hence making it possible to identify barriers to attaining the goals as well as providing actions. Solutions could be regular training, group discussions, and debriefings with feedback of sub-optimal treatment cases. Improvement suggestions can emerge during these conversations. Cruz et al. related that the protocol underwent serial reviews to incorporate suggested modifications, which increased efficiency and empowered staff (12).

Rapid recognition of sepsis through standardized screening and procedures to guide the management of patients identified as at-risk for sepsis should be an essential component of sepsis QI programs.

Han et al. showed an association between early implementation of clinical practices consistent with the 2002 guidelines in a community hospital improved outcomes in newborns and children (mortality rate 8 vs. 38%). Every hour that went by without restoration of normal blood pressure for age and capillary refill <3 s was associated with a two-fold increase in adjusted mortality odds ratio (3).

Paul et al. implemented a hospital-wide QI initiative to improve compliance with all five elements of the ACCM/PALS guidelines first-hour recommendations: (1) recognition, (2) establishing IV access, (3) starting IV fluids and resuscitation as needed, (4) administering antibiotics, and (5) starting vasoactive agents if needed (17).

### Venous Access

It is known that obtaining venous access in the pediatric age group is difficult, especially in perfusion altered states, and it is a great barrier that surrounds delay of fluid administration and other therapies. Oliveira et al. and Cruz et al. also described this adherence barrier to guidelines (12, 30). Oliveira et al. observed that in many cases, the venous line access was not adequate for high infusions rates (30). Training the team to obtain intraosseous access and the staff to get peripheral and central accesses guided by ultrasound can be the solution for better performance (31).

### Antimicrobials

Regarding antimicrobials, the members of SSC 2020 recommend starting therapy as soon as possible, preferably within 1 h of recognition. Cultures should be collected prior to antimicrobial administration but should not delay initiation of therapy (9). In this context, the awareness of physicians, pharmacists, and nurses is very important to prescribe antimicrobials that can be scheduled within the first hour in cases of septic shock. The involvement of a clinical pharmacist is also very important for improvement of antibiotics timing, as reported in a study of Cruz et al. (12).

### Vasoactive Drugs

The 2020 SSC recommends that all vasoactive agents (including norepinephrine) may be initiated through peripheral venous (or intraosseous, if in place) access if central venous access is not readily available to avoid delays in therapy (9).

Ventura et al. tested the 2007 vasoactive drug recommendation in a randomized trial and found that the use of peripheral adrenaline infusion reduced mortality to 7% compared with 20% with use of peripheral dopamine infusion until central access was secured (32).

But the indication to start vasoactive drugs earlier is still a barrier to treatment.

Kessler et al. applied standardized, *in situ*, simulations to measure and compare adherence to pediatric sepsis guidelines across 24 EDs in USA. They observed that in the first 15 min of diagnosis, all teams administered high flow oxygen, 87% of teams established first intravenous or intra osseous access, 55% of teams administered 60 ml/kg of fluids, and 62% of teams started a vasopressor after the third bolus (33). Training in simulated scenarios could be one solution (13).

### Working Conditions

The lack of adequate staff is a barrier pointed by Cruz et al. and Kortz et al. during process of sepsis protocol implementation (12, 34).

Workload during pediatric emergencies is not well-studied. Tofil et al. applied the National Aeronautics and Space Administration-Task Load Index on sepsis scenarios in nine pediatric simulation centers (United States, Canada, and United Kingdom). The researchers concluded that the team leader and team members were under moderate workloads during a pediatric sepsis scenario. The team leader's workload was high (>60) in the mental demand and effort subscales. The researchers also suggest that decreasing team leader responsibilities may improve team workload distribution (35).

In this way, QI projects help to re-think the process and optimize staff tasks. Also, protocols should help to identify severe patients and better allocate resources (12).

### Healthcare Staff Reluctance

The engagement of healthcare professionals is the core of a successful sepsis protocol implementation.

Cabana et al. conducted a systematic review and didactically resumed barriers to physician adherence to practice guidelines. According to them, the behavior change begins with knowledge and attitude. Barriers to knowledge are lack of familiarity and awareness. Barriers to attitudes are lack of agreement with specific guidelines, lack of outcome expectancy, lack of self-efficacy, and lack of motivation. Barriers to behavior are as follows: external barriers, factors related to guideline, and environmental factors (36).

Leadership, education, and feedback might help on engagement of healthcare professionals.

Swensen discussed on IHI White paper entitled High-Impact leadership that there is solid evidence that leadership engagement and focus drives improvements on health care quality and reduces patient harm. Also, high-impact leadership is not just for senior leaders but is required at every level of care delivery organization. The IHI High-Impact Leadership Framework is centered in persons and community. The leader should create a vision and build will, develop capability, and deliver results. The consequence of all efforts is the change of institutional culture (“Shape Culture”) and engagement across boundaries (37).

Examples of educational strategies are the following. Fernandez-Sarmiento et al. applied an education intervention reinforcing and updating concepts of sepsis. Emergence department professionals received a 40-min training that included lectures and case-based learning with a test before and after educational strategy (2). The education strategy of Long et al. was in the form of sepsis workshops, presentations, bedside teaching, and simulation-based initiatives (14).

Feedback has been described as good tool to improve adherence of patient and healthcare professionals to many diseases' protocols such as asthma, tuberculosis, kidney transplant, and patient safety (38–41). In the study by Schramm et al., the protocol adherence rate increased from 12.7 to 53.7% after weekly feedbacks (42). Larsen et al. applied feedback in monthly ED meetings (20). In study of Cruz et al., feedback was important to improved processes such as additional medications that were commonly used in patients with shock availability, prompt laboratory results, and changing the empiric antibiotics choice for previously healthy children (12). Feedback happens by sharing results, discussion groups, and regular meetings. The team would be motivated if they perceived that they are saving lives with protocolized treatment.

We summarized the barriers to protocol implementation and possible solutions on Table 1.

**Table 1:** Barriers and solutions to protocol implementation.

**Barriers**	**Solutions**
Parental knowledge	Education
Diagnoses by healthcare professionals	Institutional protocol, standardized screening
Venous access	Ultrasound usage, intraosseous access
Antimicrobials	Engagement of clinical pharmacist
Vasoactive drugs	Training in simulated scenarios
Working conditions	Decrease team leader responsibilities, improve the team's workload distribution, QI projects, and protocols usage
Healthcare staff reluctance	Leadership, feedback, education

## Challenges in Low-Middle-Income Countries

The 2020 SSC were developed not taking into account the healthcare resources involved and the guidelines constitute a general “best practice.”

The choice of protocol to be implemented depends on the institution. In Latin America, for example, the protocol of the Latin American Sepsis Institute (ILAS) is available, with all the established implementation guidelines (43).

Some causes of sepsis and resources of treatment may differ in Low-Middle-Income Countries (LMIC) from high-income countries. We recognize that some studies developed in LMIC evaluating a sepsis protocol implementation showed different results compared to high income countries. Maitland et al. reported an increase in mortality after fluids bolus in African children with severe infection (44). Studies by Kortz et al. (Tanzania) and Vekaria-Hirani et al. (Naroby) did not observe reduction on mortality after a protocol implementation (34, 45). Kortz et al. even observed an increase in length of stay after protocol implementation caused by complications of treatment (34). On the other hand, de Oliveira et al. (Brazil) showed that using the ACCM/PALS guidelines associated with ScvO (2) goal-directed therapy improved mortality and new organ dysfunction in children with severe sepsis (46).

Hence, while we acknowledge that following the steps proposed by the Survival Sepsis Campaign are of extreme importance, the findings above re-enforce the fact that each protocol can be personalized to a certain degree locally, according to the most frequent diseases, disposable resources, scientific evidence and training, and expertise of the healthcare professionals. Local protocols must take into account the physiopathology of the most prevalent causes of septic shock in that population.

## Protocolized or Personalized Treatment?

Protocols recommend the same treatment to all patient populations to improve treatment and allow for the most important parts of the therapy to be performed reliably at a population care level. In the individual care, studies suggest that different genetic profiles are related to different morbidity and mortality in sepsis and septic shock, possibly with different inflammation mechanisms and physiopathology (47–52). These findings suggest that a personalized approach, in addition to the protocolized treatment, may be available in the future treatment of sepsis, with earlier identification of genetic/receptors variants and targeted treatments.

## Conclusions

We resumed this review with some clues to help on successful implementation of pediatric sepsis protocols.

1) Be simple
The protocols of studies cited on our revision had five topics that work on points of fragilities of the process.
2) Be precise
Quality and Improvement initiatives are important on design the process and diagnosis of barriers
3) Educate
Education is important to keep the team updated and aware for sepsis diagnosis and treatment
4) Work as a team
Multidisciplinary teams are important for successful implementation in many hospitals
5) Communicate and celebrate results
Let the team know results of adherence and mortality.

## Author Contributions

All authors listed have made a substantial, direct and intellectual contribution to the work, and approved it for publication.

## Conflict of Interest

The authors declare that the research was conducted in the absence of any commercial or financial relationships that could be construed as a potential conflict of interest.

## Publisher's Note

All claims expressed in this article are solely those of the authors and do not necessarily represent those of their affiliated organizations, or those of the publisher, the editors and the reviewers. Any product that may be evaluated in this article, or claim that may be made by its manufacturer, is not guaranteed or endorsed by the publisher.
